# European cardiovascular magnetic resonance (EuroCMR) registry – multi national results from 57 centers in 15 countries

**DOI:** 10.1186/1532-429X-15-9

**Published:** 2013-01-18

**Authors:** Oliver Bruder, Anja Wagner, Massimo Lombardi, Jürg Schwitter, Albert van Rossum, Günter Pilz, Detlev Nothnagel, Henning Steen, Steffen Petersen, Eike Nagel, Sanjay Prasad, Julia Schumm, Simon Greulich, Alessandro Cagnolo, Pierre Monney, Christina C Deluigi, Thorsten Dill, Herbert Frank, Georg Sabin, Steffen Schneider, Heiko Mahrholdt

**Affiliations:** 1 Department of Cardiology and Angiology, Contilia Heart and Vascular Center, Essen, Germany; 2 Comprehensive Cardiology of Stamford and Greenwich, Stamford, CT, USA; 3 C.N.R./Regione Toscana “G. Monasterio Foundation”, Pisa, Italy; 4 Cardiac MR Centre, University Hospital (CHUV), Lausanne, Switzerland; 5 Department of Cardiology, VU Medical Centre, Amsterdam, The Netherlands; 6 Department of Cardiology, Hospital Agatharied, Hausham, Germany; 7 Department of Cardiology, Klinikum Ludwigsburg, Germany; 8 Department of Cardiology, University of Heidelberg, Heidelberg, Germany; 9 Barts and The London NIHR Biomedical Research Unit, The London Chest Hospital, London, UK; 10 King’s College London BHF Centre of Excellence, Division of Imaging Sciences, NIHR Biomedical Research Centre at Guy’s and St.Thomas’ NHS Trust Foundation, The Rayne Institute, St. Thomas’ Hospital, London, UK; 11 CMR Unit, Royal Brompton Hospital, Sydney Street, London, SW3 6NP, UK; 12 Department of Cardiology, Robert Bosch Medical Center, Auerbachstrasse 110, 70376, Stuttgart, Germany; 13 Department of Internal Medicine, Krankenhaus Benrath, Düsseldorf, Germany; 14 Department of Internal Medicine and Cardiology, Donauklinikum Tulln, Austria; 15 Institut für Herzinfarktforschung, Ludwigshafen, Germany

**Keywords:** Cardiovascular magnetic resonance, Registry, Quality, Safety, Therapeutic implications, Impact, Patient management

## Abstract

**Abstract:**

**Background**

The EuroCMR registry sought to evaluate indications, image quality, safety and impact on patient management of clinical routine CMR in a multi-national European setting. Furthermore, interim analysis of the specific protocols should underscore the prognostic potential of CMR.

**Methods**

Multi-center registry with consecutive enrolment of patients in 57 centers in 15 countries. More than 27000 consecutive patients were enrolled.

**Results**

The most important indications were risk stratification in suspected CAD/Ischemia (34.2%), workup of myocarditis/cardiomyopathies (32.2%), as well as assessment of viability (14.6%). Image quality was diagnostic in more than 98% of cases. Severe complications occurred in 0.026%, always associated with stress testing. No patient died during or due to CMR. In 61.8% CMR findings impacted on patient management. Importantly, in nearly 8.7% the final diagnosis based on CMR was different to the diagnosis before CMR, leading to a complete change in management. Interim analysis of suspected CAD and risk stratification in HCM specific protocols revealed a low rate of adverse events for suspected CAD patients with normal stress CMR (1.0% per year), and for HCM patients without LGE (2.7% per year).

**Conclusion**

The most important indications in Europe are risk stratification in suspected CAD/Ischemia, work-up of myocarditis and cardiomyopathies, as well as assessment of viability. CMR imaging is a safe procedure, has diagnostic image quality in more than 98% of cases, and its results have strong impact on patient management. Interim analyses of the specific protocols underscore the prognostic value of clinical routine CMR in CAD and HCM.

**Condensed abstract:**

The EuroCMR registry sought to evaluate indications, image quality, safety and impact on patient management of clinical routine CMR in a multi-national European setting in a large number of cases (n > 27000). Based on our data CMR is frequently performed in European daily clinical routine. The most important indications in Europe are risk stratification in suspected CAD/Ischemia, work-up of myocarditis and cardiomyopathies, as well as assessment of viability. CMR imaging is a safe procedure, has diagnostic image quality in more than 98% of cases, and its results have strong impact on patient management. Interim analyses of the specific protocols underscore the prognostic value of clinical routine CMR in CAD and HCM.

## Background

The German pilot phase of the EuroCMR Registry concluded on the basis of 11040 consecutive patients from 20 German centers that CMR is frequently performed in German clinical practice [1]. The most important indications in Germany were work-up of myocarditis/cardiomyopathies, risk stratification in suspected CAD/Ischemia, and assessment of viability. Furthermore, the registry data indicated that CMR imaging as used in the centers of the German pilot registry was a safe procedure, had diagnostic image quality in 98% of cases, and its results had strong impact on patient management.

In the meantime more than 27000 consecutive patients from 57 European centers in 15 countries have been included in the EuroCMR Registry. With the current analysis we sought to evaluate indications, image quality, safety, and impact on patient management of routine CMR imaging on a European level. Specifically we aim to demonstrate that the results of the German pilot data [1] hold true in this much larger multi-national and multi-ethnical population. In addition, interim analysis of the specific protocols initiated on the basis of the German pilot data should underscore the prognostic potential of CMR in certain indications, such as risk stratification in suspected CAD and hypertrophic cardiomyopathy [1,2] in a multi-national clinical routine setting.

## Methods

### Study population and data management

**Figure 1:**
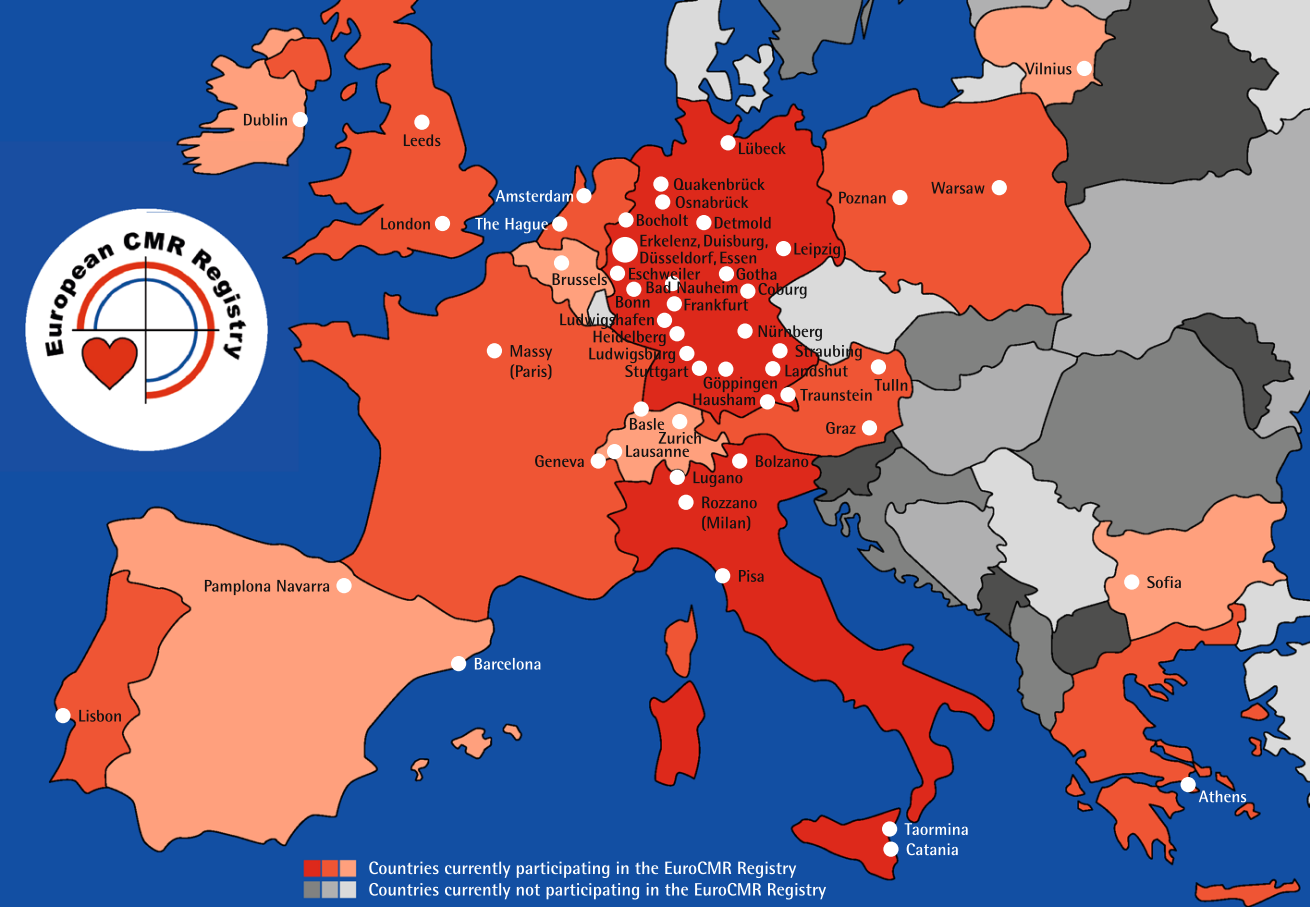
Political map of Europe visualizing all 57 participating centers in 15 countries.

The basis of the current manuscript is the EuroCMR registry. This registry includes 27781 consecutive CMR scans from 27301 consecutive patients undergoing CMR according to the ACCF/ACR/SCCT/SCMR/ASNC/NASCI/SCAI/SIR consensus appropriateness criteria for CMR imaging [3] in 57 participating sites in 15 European countries (Figure 1). All procedures were in compliance with the standardized SCMR recommended protocols [4]. All data were prospectively collected by trained personnel, manually entered in online case record forms provided by the “Institut für Herzinfarktforschung”, University of Heidelberg, Germany (http://www.herzinfarktforschung.de) via a SSL-secured internet connection, and stored on a central server. Each participating center appointed a senior investigator (either SCMR or EuroCMR level 3 trained, or licensed for CMR by the local chamber of physicians, which e.g. in Germany has stricter requirements than SCMR level 3 (two years full time training)) as local investigator responsible for data quality of each patient entered. If necessary, this local investigator contacted all sources of information necessary in order to determine more complex variables, such as the impact of CMR on patient management. A plausibility check was carried out after submitting the data to minimize further queries. Benchmarking reports were regularly made available to the local investigators for quality control. Local ethics committees approved data collection and management for every center.

### Analysis cohort

All 27781 CMR scans enrolled until June 2012 were included in the analysis. The completeness of the analysis dataset was higher than 98%. For some types of analysis the cohort was divided in patients that underwent CMR stress testing (n = 10228, including patients with suspected as well as with known CAD), and patients that did not undergo stress CMR (n = 17136). Some patients were also part of a previous report [1].

### Variables, definitions and endpoints

All variables assessed were pre-defined, and were collected directly from patients, and/or from medical records. Variables include anonymized demographic data, history, indication for CMR, procedural parameters, complications, results of CMR, the impact of CMR on clinical management, as well as clinical follow-up data for patients included in the specific protocols. Many fields are self-explanatory, all other fields (including follow-up end points for specific protocols) are defined and described in previous publications [1,2]. In case of a suspected event [2], all necessary medical records were obtained and reviewed by members of the steering committee acting as endpoint committee.

### Statistics

Since the objectives of this registry are descriptive in nature, no formal hypothesis testing was done. Absolute numbers and percentages were computed to describe the patient population. Medians (with quartiles) or means (with standard deviation) were computed as appropriate. Categorical values were compared by chi-square test or Fisher’s exact test and continuous variables were compared by two-tailed Wilcoxon rank sum test. The Cochrane-Armitage test was used for analyzing trends regarding age groups. P-values <0.05 were considered significant. Kaplan Meier curves were calculated for visualizing the cumulative survival free of events of patients with normal and several abnormal CMR results. Log-rank tests were performed to compare survival curves. All p-values were results of two-tailed tests. The tests were performed using the SAS© statistical package, version 9.1 (SAS, Cary, North Carolina).

## Results

### General use of CMR in the European clinical routine and most important indications

The most important indications for CMR in Europe were 1) risk stratification in suspected CAD/Ischemia in known CAD (34.2%), 2) work-up of myocarditis and cardiomyopathies (32.2%), followed by 3) the assessment of myocardial viability (14.6%). Ninety-two percent of all patients received a gadolinium based contrast agent. Baseline characteristics can be viewed in Table 1.

**Table 1:** Baseline characteristics

**N or quartiles**		
All	100%	27781
Male	65.5%	17841/27249
Female	34.5%	9408/27249
Age (yrs)	60.0	47.0 - 70.0
BMI (kg/m^2^)	26.2	23.7 - 29.3
Field		
1.0-T	0.5%	134/27669
1.5-T	93.6%	25899
3.0-T	5.9%	1636
Stress		
No stress	62.6%	17158/27395
Adenosine	29.3%	8018
Dobutamine	8.1%	2219
Reader		
Cardiologist	70.7%	19589/27703
Team of cardiologist and radiologist	26.7%	7398
Radiologist	2.6%	716
Primary indication for CMR		
Myocarditis/cardiomyopathies	32.2%	8950/27767
Suspected CAD/ischemia in known CAD	34.2%	9508
Myocardial viability	14.6%	4048
Valvular heart disease	5.4%	1495
Aortic disease	3.7%	1026
Congential heart disease	2.2%	624
Ventricular thrombus	1.2%	330
Cardiac masses	1.0%	288
Pulmonary vessels	1.0%	282
Coronary vessels	0.2%	57
Other than above	10.7%	2963

Values are **%** (n) or mean (standard deviation).

*BMI* Body mass Index.

### Procedural safety in the European clinical routine

Nearly 97% of all CMR procedures (n = 27781) were performed without complications. Mild complications occurred in 3.6% of patients (n = 994), and severe complications in 0.026% only (n = 7). In the group with mild complications most events (e.g. dyspnea, chest pain, extra systoles, etc.) occurred during dobutamine or adenosine infusion (75%), followed by mild allergic reactions after injection of contrast (e.g. mild urticaria or exanthema) in 5% of cases.

In the 7 patients with severe complications we found non-sustained VT (n = 2) and ventricular fibrillation (n = 1) during dobutamine infusion, as well as overt heart failure (n = 2), unstable angina (n = 1), and anaphylactic shock (n = 1) in the setting of adenosine stress. All severe complications were related to stress testing (Table 2). Procedural safety was not dependent on gender, age of the patient, the country or the center the scan was performed.

**Table 2:** Complications related to no stress vs. stress CMR

	**All (n = 27396)**		**No stress (n = 17136)**		**Stress (n = 10228)**	
Complications						
None	96.3%	(n = 26395)	98.6%	(n = 16893)	92.6%	(n = 9476)
Mild	3.6%	(n = 994)	1.4%	(n = 243)	7.3%	(n = 745)
Severe	0.0%	(n = 7)	0.0%	(n = 0)	0.1%	(n = 7)

Values are **%** (n).

### Image quality in the European clinical routine

Good image quality was achieved in 88.0% (n = 24094) of patients. In 10.3% (n = 2817) image quality was moderate but still diagnostic. Poor image quality (non-diagnostic) was present in 1.7% of patients only (n = 451). No relevant difference was found comparing stress to no stress CMR (good image quality in 87.2% without stress vs. 89.5% with stress).

Image quality was not dependent on gender of the patients, nor the country or the center the scan was performed. However, there was a significant trend towards poorer image quality in older patients (>75 yrs vs. <45 yrs; p < 0.0001). Despite this decrease of image quality with age, the ability of CMR to derive a diagnosis and the impact on patient management was not affected (Table 3). In fact, the percentage of therapeutic consequences was even higher in older patients compared to younger patients (>75 yrs vs. <45 yrs; p < 0.0001).

**Table 3:** Indications, image quality, and complications related to patient age

	**≤44 yrs**	**45 – 59 yrs**	**60 – 74 yrs**	**≥75 yrs**
Indication				
Ischemia/CAD	12.1%	37.7%	48.1%	49.5%
Myocarditis/CMP	63.6%	36.6%	22.5%	16.5%
Viability	5.3%	17.0%	19.2%	22.4%
Stress CMR	13.7%	38.0%	47.5%	47.9%
Image quality				
Good	92.6%	90.8%	86.2%	80.1%
Moderate	6.3%	8.0%	11.9%	16.9%
Poor	1.1%	1.2%	1.9%	3.0%
Complications				
None	98.4%	96.7%	95.5%	94.4%
Mild	1.6%	3.3%	4.5%	5.5%
Severe	0.0%	0.1%	0.0%	0.0%
New diagnosis	9.3%	9.5%	8.7%	7.7%
Therapeutic consequence	40.4%	51.9%	58.4%	64.6%

### Impact of CMR on patient management in the European clinical routine

In nearly two thirds of all patients (61.8%) we could demonstrate direct impact of CMR on the clinical management by providing an unsuspected new diagnosis (8.7%) and/or resulting in therapeutic consequences as described in Table 4. Table 5 demonstrates the impact on patient management by indication for the three most common CMR indications as described above.

**Table 4:** Impact of CMR on patient management

		**N or quartiles**
All	100%	27781
Completely new diagnosis not suspected before	8.7%	2354/27006
Therapeutic consequences		
Change in medication	25.0%	6689/26743
Invasive procedure	16.8%	4510/26778
Hospital discharge	10.2%	2738/26771
Hospital admission	1.4%	386/26780
Impact on patient management (new diagnosis and/or therapeutic consequence)	61.8%	16677/27006

Values are **%** (n).

**Table 5:** Impact of CMR on patient management by indication

	**Myocarditis/CMP**	**Suspected CAD/Ischemia**	**Viability**
All (from n = 27781)	32.2%	34.2%	14.6%
Completely new diagnosis not suspected before	11.4%	8.1%	5.3%
Therapeutic consequences			
Change in medication	25.3%	24.3%	33.2%
Invasive procedure	6.9%	23.1%	24.2%
Hospital discharge	10.4%	14.3%	6.9%
Hospital admission	1.1%	1.5%	1.9%
Impact on patient management (new diagnosis and/or therapeutic consequence)	55.1%	71.4%	71.5%

Focusing on the group of patients that underwent stress CMR for work-up of suspected CAD or suspected ischemia in known CAD reveals that in nearly half the cases (45%) invasive angiography could be avoided based on the results of CMR (Table 6).

**Table 6:** Additional diagnostic procedures avoided due to results of CMR

	**All (n = 27025)**		**No stress (n = 16526)**		**Stress (n = 10113)**	
Invasive angiography	24%	(n = 6483)	11.6%	(n = 1921)	45%	(n = 4555)
Nuclear (SPECT/PET)	20.6%	(n = 5574)	9.8%	(n = 1624)	39%	(n = 3946)
Coronary CT	11.8%	(n = 3182)	5.9%	(n = 976)	21.8%	(n = 2202)

Values are % (n).

*PET* Positron emission tomography.

### Interim analysis of the first two specific protocols

#### Specific protocol - suspected CAD

The main aim of this specific EuroCMR Registry protocol is to demonstrate that patients presenting for workup of suspected CAD, which have a completely normal CMR scan, will have a low risk for adverse cardiovascular events during follow-up in a multi-national clinical routine setting. A detailed description of this protocol including the definitions of variables and adverse events has been published previously [2].

**Figure 2:**
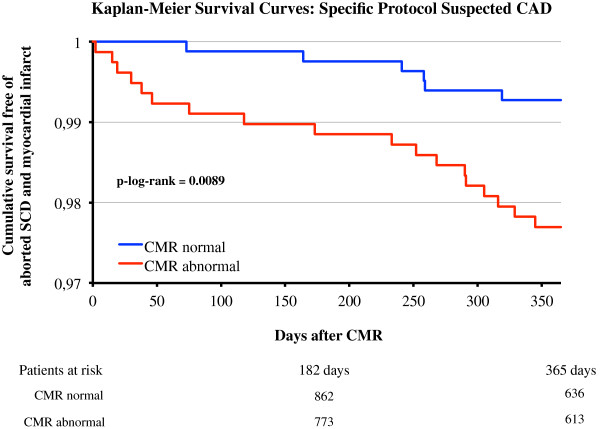
**Kaplan-Meier Survival Curves for the specific protocol “suspected CAD” with regard to death, aborted SCD and non-fatal myocardial infarction. **The number of patients at risk is shown at the bottom of the figure.

At the end of June 2012 more than 3300 patients have been enrolled in this specific protocol, which is still open for ongoing recruitment. Clinical follow-up data (mean 400d, IQR 367d-419d, follow-up rate 90%) is currently available for 1706 patients. In the subgroup of patients with normal CMR (n = 866, defined as LV-EF ≥ 60% and LV-EDV ≤ 180 ml and no ischemia and no LGE) the rate of major adverse events (all cause death, aborted SCD, or non-fatal myocardial infarct [2]) was 1.0% per year during follow-up. In the group with abnormal CMR (n = 840, defined as LV-EF < 60% or LV-EDV > 180 ml or ischemia or LGE) the event rate was 2.7% per year, see Figure 2.

#### Specific protocol - risk stratification in HCM

The main aim of this specific EuroCMR Registry protocol is to establish the role of LGE in risk stratification of HCM patients with regard to cardiac death. Specifically we sought to confirm that the presence of LGE is an independent risk factor for cardiac death and other adverse events in HCM patients. A detailed description of this protocol has also been published previously [2].

**Figure 3:**
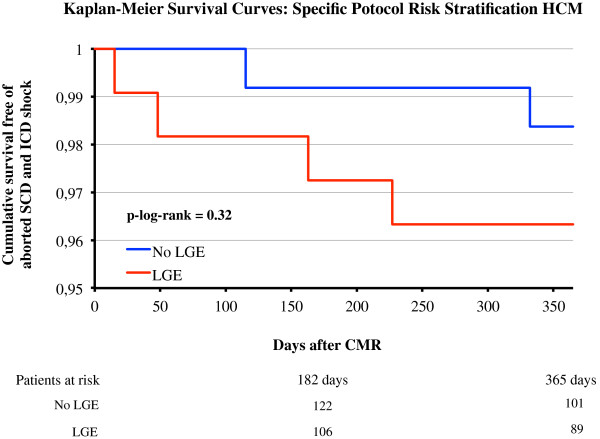
**Kaplan-Meier Survival Curves for the specific protocol “risk stratification HCM” with regard to death, aborted SCD and adequate ICD discharge. **The number of patients at risk is shown at the bottom of the figure.

At the end of June 2012 more than 550 patients have been enrolled in this specific protocol, which is also still open for ongoing recruitment. Clinical follow-up data (mean 409d, IQR 372d-437d, follow-up rate 90%) is currently available for 249 patients. In the subgroup of patients without LGE (n = 115) the rate of major adverse events (all cause death, aborted SCD, or adequate ICD discharge [2]) was 2.2% per year during follow-up. In the group with any LGE present in the myocardium (n = 134), the event rate was 4.3% per year, see Figure 3.

## Discussion

This dataset is unique in that it describes the clinical use, including indications, image quality, procedural safety and impact on patient management of CMR in more than 27000 patients from 57 centers in 15 countries (see Additional file 1: Data supplement). Our data confirm pilot results [1] indicating that CMR is frequently performed in clinical routine, is a safe procedure, has diagnostic image quality in more than 98% of cases, and its results have strong impact on patient management. Furthermore, interim analyses underscore the prognostic potential of CMR in the clinical routine.

### General use of CMR in the European clinical routine and most important indications

Similar to our pilot data, more than 92% of CMR procedures involved the use of Gadolinium based contrast media, allowing the detection of small subendocardial infarcts [5,6], prediction of recovery of ventricular function before revascularisation [7], risk stratification in suspected coronary artery disease [8,9], evaluation of myocardial ischemia [10], as well as assessment of cardiomyopathies [11], and myocarditis [12-14], respectively.

In line with pilot phase results [1], CMR case reading and reporting was done by cardiologists (70.7%), a team of cardiologists and radiologists (26.7%), or radiologists alone (2.6%). This finding may be influenced by a selection bias, since the registry was initiated and is run by a cardiologist society. However, we did not find significant differences in image quality, safety or impact on patient management comparing those three reading/reporting groups.

In comparison to the pilot data however, the clinical routine use of CMR imaging at 3 Tesla increased from 0.8% to 5.9% in the current dataset. This may be explained by advantages of 3 Tesla compared to 1.5 Tesla, such as improved speed (e.g. CMR perfusion), and/or spatial resolution [15,16].

### Procedural safety in the European clinical routine

Mild complications occurred in 3.6%, and severe complications in 0.026% of patients only. No patient died during or due to CMR, confirming that CMR is safe when performed in a multi-national routine clinical setting. All severe complications were related to stress testing (Table 2), in line with the German pilot results [1]. Importantly, the procedural safety of CMR is not dependent on race, gender or age of patients, despite a much greater racial diversity in the current European dataset.

However, one important limitation of our data concerning CMR safety may be that not all EuroCMR Registry patients undergo systematic clinical follow-up (specific protocols only, [2]) and thus, theoretically possible cases of NSF may have been missed. However, we did not receive any reports of NSF from the patients undergoing clinical follow-up in one of the specific protocols. Nevertheless, serum creatinine and glomerular filtration rate should be evaluated and taken into account prior to any gadolinium contrast administration.

Since most complications of stress CMR are not related to CMR imaging itself, but to stressing the patient, stress CMR has been confirmed to be at least as safe as stress echocardiography [17], stress nuclear testing [18], or even as safe as obtaining a simple treadmill ECG (about one fatal complication or myocardial infarct in 2500 cases) [19].

### Image quality in the European clinical routine

To our knowledge, this is the first dataset on clinical routine image quality of CMR in a European setting. Our data demonstrate that CMR is capable of answering the relevant clinical questions in more than 98% of cases. This indicates, that current CMR utilisation yields a high number of valuable studies, most probably related to the good image quality. Only 1.7% of studies were inadequate in quality, allowing no diagnosis.

Importantly, this was shown in a multi-national, multi-ethnical consecutive clinical routine setting, including all comers such as patients with dyspnea at rest, atrial fibrillation, obesity (body mass index quartiles 23.7-29.3 kg/m^2^), or other frequent cardiac conditions affecting image quality. Thus, the average image quality of CMR in the clinical routine is better than the average image quality of other non-invasive imaging techniques, such as echocardiography [20], cardiac CT [21,22], or SPECT [23]. In addition, no ionising radiation is needed for CMR, which can therefore be repeated as often as necessary for follow-up purposes.

Matching our German pilot data [1], we found a significant decrease of image quality in older patients, which again was associated with an increased impact on patient management in this group (Table 3). This can be explained by the higher morbidity in older patients causing more gating or breathing problems, but on the other hand yielding more abnormal findings requiring an altered management (Table 3).

### Impact of CMR on patient management in the European clinical routine

CMR had direct impact on the clinical management of the majority of patients (Tables 4 and 5), also confirming the earlier German pilot results [1] in the European clinical routine. In patients undergoing CMR stress testing for work-up of CAD (Table 6) invasive angiography could be avoided in nearly half the patients (n = 4555), underscoring the role of CMR stress testing as a gatekeeper for invasive angiography. In addition, nearly 6148 non-invasive procedures involving the use of ionizing radiation, such as SPECT imaging could also be avoided on the basis of the CMR results (Table 6).

We did not yet perform a cost analysis of integrating CMR into the clinical routine at a European level. However, a cost effectiveness analysis performed on the basis of the German pilot data [1] indicates that integrating CMR in the clinical routine does not increase the overall costs of patient care, but reduces costs between 11% and 65% in most cases [24].

### Interim analysis of the first two specific protocols

#### Specific protocol - suspected CAD

Our current interim data indicate a rate of major adverse events (all cause death, aborted SCD, or non-fatal myocardial infarct [2]) of 1.0% per year for patients with normal stress CMR in the group of 1706 patients that underwent 12-month follow-up in this specific protocol so far (Figure 2). This confirms the results of earlier controlled studies in smaller (often single-center) populations [25-27] in a multi-national and multi-ethnical clinical routine setting, underscoring the prognostic value of clinical routine CMR for this indication.

#### Specific protocol - risk stratification in HCM

On the basis of 249 patients that have undergone 12-month follow-up so far, we already found a trend towards better outcome in HCM patients without LGE (Figure 3), also confirming the results of earlier controlled single center studies [28,29] in a multi-national clinical routine setting, and almost exactly matching the calculated event rates of a recent meta analysis [30]. However, in the studies mentioned above the mean follow-up time was much longer (median more that 3 years) then in this registry protocol so far (just 12-month), which most likely explains why the trend in the registry data does not yet reach statistical significance.

## Conclusion

The current EuroCMR Registry data including more than 27000 patients from 57 centers in 15 countries confirm that CMR is frequently performed in European daily clinical practice. The most important indications in Europe are risk stratification in suspected CAD/Ischemia, work-up of myocarditis and cardiomyopathies, as well as assessment of myocardial viability. CMR imaging as used in the centres of the EuroCMR Registry, is a safe procedure, has diagnostic image quality in more than 98% of cases, and its results have strong impact on patient management. Interim analyses of the specific protocols underscore the prognostic value of clinical routine CMR in HCM and suspected CAD in a multi-national clinical routine setting.

## Abbreviations

CAD: Coronary artery disease; CHF: Congestive heart failure; CMR: Cardiovascular magnetic resonance; HCM: Hypertrophic cardiomyopathy; LGE: Late gadolinium enhancement; NSF: Nephrogenic systemic fibrosis; SCD: Sudden cardiac death; SPECT: Single photon emission tomography; VT: Ventricular tachycardia.

## Competing interests

The EuroCMR Registry is supported by unrestricted educational grants from the following companies (in alphabetic order): ●Medtronic Inc., Minneapolis MN, USA. ●Novartis International AG, Basel, Switzerland. ●Siemens Health Care, Erlangen, Germany. Importantly, industry sponsoring was exclusively used for registry data management and analysis. All CMR scans reported in this registry were clinically indicated according to the actual appropriateness criteria [3], and thus completely funded by the regular health care providers.

## Authors’ contributions

OB and AW contributed to the idea and design of the study, recruited the patients, acquired and analyzed the data, and wrote the report. ML, JS, AR, GP, DN, HS, SP, EN, SP, JS, SG, AC, PM, CD, TD, HF, GS, and SS contributed to the idea and design of the study, analysis of the data, and revision of the report. HM designed the study, contributed to the acquisition and analysis of the data, and wrote the report. All authors read and approved the final manuscript.

## Supplementary Material

Additional file 1 Data supplement. Click here for file
