# Supplementary material for: European cardiovascular magnetic resonance (EuroCMR) registry – multi national results from 57 centers in 15 countries
Source: J Cardiovasc Magn Reson. 2013 Jan 18;15(1):9. doi: 10.1186/1532-429X-15-9 (PMC3564740; doi:10.1186/1532-429X-15-9)
Supplement: Additional file 1 — Data supplement. [file 1532-429X-15-9-S1.docx]

| **#** | **Center** |
| --- | --- |
| 1 | Elisabeth Hospital, Essen, Germany |
| 2 | Robert Bosch Medical Center, Stuttgart, Germany |
| 3 | Hospital Agatharied, Hausham, Germany |
| 4 | Hospital Ludwigsburg, Germany |
| 5 | University Hospital of Heidelberg, Germany |
| 6 | Heart center, Ludwigshafen, Germany |
| 7 | Hospital Coburg, Germany |
| 8 | Hospital Benrath, Düsseldorf, Germany |
| 9 | Hospital Traunstein, Germany |
| 10 | Marien Hospital, Osnabrück, Germany |
| 11 | Hermann Josef Hospital, Erkelenz, Germany |
| 12 | Cardiological Center Bethanien, Frankfurt am Main, Germany |
| 13 | Hospital Achdorf-Landshut, Landshut, Germany |
| 14 | Christian Hospital Quakenbrück, Germany |
| 15 | St. Antonius Hospital Eschweiler, Germany |
| 16 | Hospital St. Elisabeth, Straubing, Germany |
| 17 | Helios Hospital Gotha, Germany |
| 18 | Hospital „Am Eichert“, Göppingen, Germany |
| 19 | Kerckhoff Hospital GmbH, Bad Nauheim, Germany |
| 20 | University Hospital Leipzig, AöR; Germany |
| 21 | Hospital Lippe-Detmold, Detmold, Germany |
| 22 | Hospital Nürnburg, Germany |
| 23 | Heart center Leipzig, Germany |
| 24 | Heart center Duisburg, Germany |
| 25 | Lutheran Hospital Düsseldorf, Germany |
| 26 | St. Marien Hospital Bonn, Germany |
| 27 | St. Agnes Hospital, Bocholt, Germany |
| 28 | University Hospital Schleswig Holstein, Lübeck, Germany |
| 29 | VU Medical Centre, Amsterdam, The Netherlands |
| 30 | Haga Hosptal, The Hague, The Netherlands |
| 31 | Donau Hospital Tulln, Austria |
| 32 | Cardiology Centre, Zurich, Austria |
| 33 | LKH Graz West, Graz, Austria |
| 34 | University Hospital (CHUV), Lausanne, Switzerland |
| 35 | University Hospital Basel, Switzerland |
| 36 | Cardiocentro Ticino, Lugano, Switzerland |
| 37 | Center of diagnostic Radiology, Carouge-Geneva, Switzerland |
| 38 | Clinical Physiology Institute; National Research Council, Pisa, Italy |
| 39 | Institutio Clinico Humanitas CMR Unit, Rozzano (Milano), Italy |
| 40 | Hospital San Maurizio, Bolzano, Italy |
| 41 | Hospital San Vincenzo, Taormina, Italy |
| 42 | Hospital Ferrarotto, University of Catania, Catania, Italy |
| 43 | Hospital de la Santa Creu I Sant Pau, Barcelona, Spain |
| 44 | University Hospital Navarra, Pamplona Navarra, Spain |
| 45 | University Hospital Santa Maria, Lisbon, Portugal |
| 46 | Hospital Da Luz, Radiology and Cardiology, Lisbon, Portugal |
| 47 | King´s College London/St. Thomas´ Hospital, London, United Kingdom |
| 48 | Royal Brompton Hospital, London, United Kingdom |
| 49 | Leeds General Infirmary – LIGHT, Leeds, United Kingdom |
| 50 | Institute of Cardiology, CMR Unit, Warsaw, Poland |
| 51 | Poznan University of Medical Science, Poznan, Poland |
| 52 | Universite of Louvain (UCL), Brussels, Belgium |
| 53 | Institute Hospital Jacques Cartier, Massy, France |
| 54 | St. Vincent´s University Hospital, Dublin, Ireland |
| 55 | Onassis Cardiac Surgery Center, Athens, Greece |
| 56 | University Hospital Sant Klinikos, Vilnius, Lithuania |
| 57 | Tokuda Hospital Sofia, Bulgaria |
